# Emitter Identification of Digital Modulation Transmitter Based on Nonlinearity and Modulation Distortion of Power Amplifier

**DOI:** 10.3390/s21134362

**Published:** 2021-06-25

**Authors:** Yue Chen, Xiang Chen, Yingke Lei

**Affiliations:** School of Electronic Countermeasures, National University of Defense Technology, Hefei 230000, China; chenyue@nudt.edu.cn (Y.C.); xiaxiang83@126.com (X.C.)

**Keywords:** specific emitter identification, power-amplifier nonlinearity, modulator distortion, convolutional neural network

## Abstract

Specific transmitter identification (SEI) is a technology that uses a received signal to identify to which individual radiation source the transmitted signal belongs. It can complete the identification of the signal transmitter in a non-cooperative scenario. Therefore, there are broad application prospects in the field of wireless-communication-network security, spectral resource management, and military battlefield-target communication countermeasures. This article demodulates and reconstructs a digital modulation signal to obtain a signal without modulator distortion and power-amplifier nonlinearity. Comparing the reconstructed signal with the actual received signal, the coefficient representation of the nonlinearity of the power amplifier and the distortion of the modulator can be obtained, and these coefficients can be used as the fingerprint characteristics of different transmitters through a convolutional neural network (CNN) to complete the identification of specific transmitters. The existing SEI strategy for changing the modulation parameters of a test signal is to mix part of the test signal with the training signal so that the classifier can learn the signal of which the modulation parameter was changed. This method is still data-oriented and cannot process signals for which the classifier has not been trained. It has certain limitations in practical applications. We compared the fingerprint features extracted by the method in this study with the fingerprint features extracted by the bispectral method. When SNR < 20 dB, the recognition accuracy of the bispectral method dropped rapidly. The method in this paper still achieved 86% recognition accuracy when SNR = 0 dB. When the carrier frequency of the test signal was changed, the bispectral feature failed, and the proposed method could still achieve a recognition accuracy of about 70%. When changing the test-signal baud rate, the proposed method could still achieve a classification accuracy rate of more than 70% for four different individual radiation sources when SNR = 0 dB.

## 1. Introduction

SEI is a technology that uses signal-processing methods to extract essential difference information of different transmitters from the received signal, thereby distinguishing each communication transmitter [1]. Since the beginning of the 21st century, the development of sensor networks and mobile communication technologies has also enabled the development of the information society from the electronic age to the Internet of Things era and has promoted the birth of countless communication radiation-source devices. In the civil field, the security of wireless communication systems is receiving exponential attention. According to the characteristics of the physical layer, SEI can determine whether the received signal comes from an authorized transmitter without relying on the user key. This can undoubtedly reduce security incidents caused by key theft. Therefore, it is extensively studied. In the military field, the use of received radio signals to determine from which radio station a signal comes is conducive to grasping the information initiative on the battlefield and supporting communication confrontation.

The key to SEI is finding fingerprint features that represent the difference of specific transmitters. Fingerprint features should be universal, unique, stable, and measurable in a given target set. All individual radiation sources have versatility in fingerprint features. Uniqueness shows that the fingerprint features of individuals on different radiation sources are different. The fingerprint features that need to be extracted for stability have certain antinoise interference capabilities, and the measurability shows that the individual identification of specific radiation sources can be realized in engineering. The fingerprint characteristics of a radiation source are mainly divided into transient and steady-state characteristics. An extracted feature from the signal when the emitter is started is transient, and the extracted feature from a signal that the emitter transmits stable information is steady-state. Lin Y. analyzed transient and steady-state features. He used the power spectral density method and the fractional Fourier transform method to analyze transient signals, and the line integral bispectral method to analyze steady-state signals. Results showed that the SEI method using transient signals had a higher recognition rate than that of the SEI method using steady-state signals under a high signal-to-noise ratio (SNR). However, transient features are susceptible to noise. When the SNR is low, the recognition rate rapidly drops [2] because the transient signal does not contain the interference of sending the message, and it has rich transmitter characteristics. The radio-frequency fingerprint vector extracted from a transient signal can characterize the transmitter device well, but its stability is poor, it is susceptible to noise interference, and it is difficult to obtain a sufficient transient signal in non-cooperative scenarios. This limits its applications in engineering. Due to the presence of modulation information, some features of the steady-state signal are overwhelmed, so the recognition rate of this method is low, but when the SNR is large enough, the features that are not overwhelmed by the message still have very good individual representation performance. Steady-state signals are easy to implement, and it is easy to obtain a sufficient number of steady-state signals. Therefore, steady-state characteristics have always been the main research object of scholars.

For steady-state features, statistical features in the time domain need to first be extracted from a received signal. Reising, Williams, and others extracted the instantaneous amplitude, instantaneous phase, the mean value of instantaneous frequency, variance, kurtosis, and other statistical parameters for the transient and pilot parts of different types of signals (such as GSM, WiMax, and ZigBee signals) [3,4,5,6]. Regarding the characteristics of the individual radiation source, experiments verified that the use of statistical time-domain parameter characteristics can identify individual radiation sources well. S. Deng proposed an algorithm of linear skewness and linear kurtosis based on skewness and kurtosis. For limited samples, linear moment estimation is more robust and accurate than other estimation methods are, and even better than maximum-likelihood estimation. Therefore, linear skewness and linear kurtosis are not sensitive to outliers, so non-Gaussian high-precision measurement can be achieved [7]. Research on radiation-source identification methods based on statistical time-domain characteristics is at a relatively early stage, but these methods are susceptible to noise; in this case, they are not enough to analyze non-Gaussian and nonstationary signals. Some subtle features are easily overwhelmed by noise or extracted signal features only reflect noise features. Statistical feature extraction based on high-order moments and high-order spectral parameters can provide richer amplitude and phase information, and this has strong anti-Gaussian noise capabilities, but the amount of calculation is large, so the integrated bispectral feature is often used to characterize individual radiation-source differences. Integrated one-dimensional spectral characteristics can be obtained by integrating the two-dimensional spectrum. According to different integration paths, this can be divided into the radial integrated bispectrum (RIB), axial integrated bispectrum (AIB), square integrated bispectrum (SIB), and circular integral bispectrum (CIB) [1,8,9,10,11].

Weak signals such as parasitic modulation and parasitic elements generated by the nonlinearity of radiation-source transmitter equipment are difficult to accurately analyze by time- and frequency-domain analysis methods. Researchers also applied feature extraction in the signal-transformation domain to the individual identification of communication radiation sources. This type of method attempts to observe or count the characteristics of signal parameters in other domains through various signal conversions, to distinguish between different individual radiation sources. Methods such as wavelet analysis, time-frequency analysis, empirical-mode decomposition (EMD) transformation, and intrinsic time-scale decomposition (ITD) transformation were applied to analyze the slightly different information of individual radiation sources, achieving a good individual recognition effect [1,12]. He. B. decomposed and preprocessed a received signal from EMD, ITD, or variational-mode decomposition (VMD), and then extracted the skewness and kurtosis of the decomposed signal to form a feature vector that characterized the individual signal. The ITD-based method obtained the highest recognition rate in the SEI problem, followed by EMD, and lastly the VMD decomposition method [13]. Jie proposed an SEI recognition method based on a 3D Hilbert energy spectrum and multiscale segmentation; the time-frequency energy spectrum was derived via Hilbert–Huang transform, which could be defined as a complicated curved surface in the three-dimensional space, namely, the 3D Hilbert energy spectrum. Then, via fractal theory, four features were extracted to compose the feature vector under multiscale segmentation. Lastly, the communication of the identification of 13 individual emitters was achieved by utilizing a support vector machine (SVM). When SNR > 26 dB, this method exceeded 90% correct recognition rate based on three simulation datasets [14].

The third type of radiation-feature extraction algorithm is based on the nonlinearity of transmitter hardware. Various analog components inside the communication radiation-source transmitter, such as digital to analog (DAC), power amplifier (PA), modulators, and filters exhibit a certain nonlinearity during operation [15,16,17]. Aiming at the nonlinear error introduced by DAC [18], Polak used a random Brownian bridge process to model the nonlinear behavior of the radiator transmitter’s DAC device [19]. Zhang and Liu used a memoryless polynomial model to describe the power-amplifier nonlinearity of different individual radiation sources [20,21,22], which simplified the solution of the nonlinear power-amplifier model, but insufficiently describing the memory effect of the power amplifier. To characterize the nonlinear behavior of a power amplifier with a weak memory effect [23], Liu presented a radio-frequency front-end nonlinearity estimator that performed SEI based on the knowledge of a training sequence. The algorithm provided robust identification by first using alternative degrees of nonlinearities associated with symbol amplitudes for initial estimation, and then iteratively estimating the channel coefficients and distorted transmit symbols to overcome the intersymbol interference effect [24]. Jian C. considered that a low-amplitude signal is less affected by the PA, the pulse of the channel response of the low-amplitude symbol could be estimated, and the nonlinearity of the PA could be extracted, reducing the influence of noise iteratively and through larger amplitude symbols. In other words, this method is only applicable to signals with different symbol amplitudes. Simulation results showed that this method could achieve a recognition rate of 90% under the condition of 18 dB [25].

**Figure 1:**
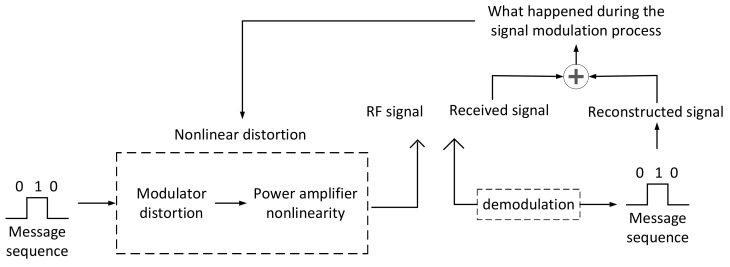
Feature-extraction flowchart.

The essential reason for the difference between radiation-source signals is that various device parameters that constitute a radiation source have a certain error range. These subtle parameter differences lead to differences in the amplitude, frequency, skewness, kurtosis, and double-order spectrum of the signal. We can distinguish a single radiation source from the surface phenomenon of the signal, but extracted features cannot characterize the difference in the internal device parameters of the radiation source. Therefore, in this paper, through the mathematical modeling of the distortion of the internal components of the radiation source and the nonlinearity of the power amplifier, parameters characterizing the distortion and nonlinearity are extracted from the received RF signal as the feature vector of different radiation sources to distinguish different radiation sources. First, the received signal was demodulated to obtain the transmitted message bit sequence, and then some basic parameters were obtained (such as carrier frequency, baud rate, modulation mode, and phase) through analysis of the signal. We could use the obtained parameters and message bit sequence to recover the signal sent by the signal sender through the signal-processing tool. The simulated signal did not pass through the transmitter. We could compare the ‘pure signal’ that we recovered without any nonlinear distortion with the actual received signal. In this way, we know what kind of nonlinear distortion the message bits experience through the transmitter. The entire process is shown in Figure 1. This method extracts the characteristics of the nonlinear parameters of the transmitter and does not change with the change in the transmitted message signal. Through processing any signal, the characteristic parameters of the hardware difference of the transmitter can be obtained, and the signal sent from the transmitter can be individually identified without being affected by the test signal carrier frequency, modulation mode, and modulation rate.

The rest of this article is arranged as follows: Section 2 first gives the nonlinear model of the power amplifier, establishes the modulator distortion models of PAM and QPSK signals, and presents the feature-extraction formulas under these two modulation modes. It also briefly introduces the traditional feature-extraction integral bispectral algorithm. Section 3 verifies the recognition performance of this method via simulation, and presents the recognition performance of pulse amplitude modulation (PAM) signal and quadrature phase-shift keying (QPSK) signals under the condition of variable carrier frequency and modulation rate. Section 4 presents the results and discussion of the experiment.

## 2. Modulator Distortion and Nonlinear Power Amplifier (PA) Model Establishment

### 2.1. Nonlinear PA Model

PA is an important part of various wireless transmitters. In the pre-stage circuit of the transmitter, the power of the RF signal generated by the modulation oscillator circuit is very small, and it needs to pass through the power amplifier to obtain sufficient RF power before it can be fed, radiating towards the antenna. When the power amplifier works in the linear-amplification region, its efficiency is relatively low, but when the power amplifier enters the saturated working region, it produces serious nonlinear distortion, which expands the signal spectrum, interferes with adjacent channels, and produces intermodulation interference [1,23].

The mathematical modeling methods of power amplifiers are mainly divided into memory and nonmemory models. When the input signal is narrow-band, a memoryless model is often used to describe the nonlinear behavior of the power amplifier, for example, the polynomial, Saleh, and RAPP models. When the input signal is wide-band, the nonlinear behavior of the power amplifier is often modeled by a memory model, such as a Volterra series model, a polynomial model with memory, a delayed neural network model, a Hammerstein model, and a Wiener model.

**Figure 2:**
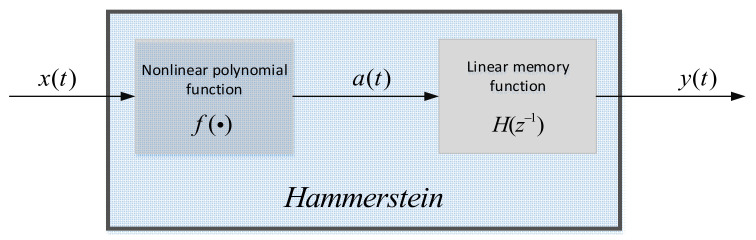
Hammerstein model.

In practical applications, even if the input is a narrow-band power-amplifier signal, there is generally a weak memory effect. Therefore, we use the linear memory (Hammerstein) model to describe the nonlinear distortion of the power amplifier. The Hammerstein models are shown in Figure 2. By introducing intermediate functions, the input–output relationship of the model can be more concise. The content shown in Figure 2 can be expressed by the following formula:

(1) $$a(t)=\sum_{i=1}^{M}\lambda_{i}{\left(x(n)\right)}^{i}$$

(2) $$y(t)=\sum_{i=1}^{D}\alpha_{i}y(n-i)+a(t)$$

where *M* is the order of the nonlinear model, $\lambda_{i}$ is the model coefficient, D is the order of the memory model, and the corresponding model coefficient is $\alpha_{i}$.

### 2.2. PAM Modulation-Distortion Model

In digital PAM, the signal waveform can be expressed as:

(3) Sm(n)=ReAmg(n)ej2πfcn=Amg(n)cos(2πfcn)

where $A_{m}$ is the amplitude of the signal, g(n) is the shaped pulse, and $f_{c}$ is the carrier frequency. In digital amplitude modulation, the distortion of the transmitter includes the following. (a) The deviation of the $A_{m}$ amplitude. When there are multiple pulse amplitudes, different radiation sources produce the same amplitude due to the allowable error of the digital-to-analog converter (DAC) and other devices. There are subtle differences. (b) The nonlinearity of the power amplifier. The radio-frequency signal is amplified by the PA, which produces nonlinear distortion. The amplitude offset produced by ΔA is represented, and we used the linear memory Hammerstein model to describe the nonlinearity of the power amplifier:

(4) x(n)=Reξ(n)ejωn=12ξ(n)ejωn+ξ∗(n)e−jωn

where $\xi (n)=\left(A_{m}+\Delta A\right)g(n)$ is the equivalent baseband complex envelope signal.

(5) $$a(n)=\sum_{i=1}^{M}\lambda_{i}{\left(x(n)\right)}^{i}=\sum_{i=1}^{M}\lambda_{i}\sum_{k=0}^{i}\frac{1}{2^{i}}C_{i}^{k}{\left(\xi (n)\right)}^{k}{\left(\xi^{*}(n)\right)}^{i-k}e^{j\omega n(2k-i)}$$

There is a frequency multiplication component in a(n), and the actual signal, except for the center frequency passband, is filtered out by the filter; therefore, 2k−i=±1. Formula (5) shows that *i* is the only term with retained odd numbers. Therefore, the equivalent low-pass of the above formula can be written as:

(6) $$a^{\prime }(n)=\sum_{i=1}^{(M-1)/2}\frac{1}{2^{2i}}C_{2i+1}^{i+1}\lambda_{2i+1}{\left(\xi (n)\right)}^{i+1}{\left(\xi^{*}(n)\right)}^{i}$$

Combined with the memory-term formula, the equivalent output of the power amplifier of the Hammerstein model is:

(7) $$y(n)=\sum_{i=1}^{D}\alpha_{i}y(n-i)+\xi (n)\sum_{i=0}^{(M-1)/2}\frac{1}{2^{2i}}C_{2i+1}^{i+1}\lambda_{2i+1}{\left|\xi (n)\right|}^{2i}$$

The above formula can be written as:

(8) y(n)=u(n)h

where u(n) is only related to y(n−i) and ξ(n), and h is only related to model parameters, not related to power amplifier input:

$$u(n)=\left[\xi (n),\xi (n){\left|\xi (n)\right|}^{2},\cdots ,\xi (n){\left|\xi (n)\right|}^{M-1},y(n-1),\cdots ,y(n-D)\right]\;h={\left[\mu_{0},\cdots ,\mu_{(M-1)/2},\alpha_{1},\cdots ,\alpha_{D}\right]}^{T}\;\mu_{i}=\frac{1}{2^{2i}}C_{2i+1}^{i+1}\lambda_{2i+1}\;\;\;i=\left(0,\cdots ,(M-1)/2\right)$$

At the receiving end, u(n) in Formula (8) is only related to the current input ξ(n) of the power amplifier and output y(n−i) of the power amplifier sometime before, where y(n−i) can be observed, and ξ(n) can be obtained by demodulating and reconstructing the signal. Therefore, u(n) is observable. Assuming the channel is additive white Gaussian noise (AWGN), Formula (8) can be written as:

(9) $$y(n)=u(n)h+\nu_{n}$$

where $\nu_{n}$ is zero-mean Gaussian white noise. For observation time steps *N*, the above formula is written in matrix form:

(10) y=Uh+V

where

$$y={\left[y(n),\cdots ,y(n-N)\right]}^{T},U=\left[\begin{array}{c} u(n)\\ \vdots \\ u(n-N) \end{array}\right]\;u(n-j)=\left[\varepsilon_{0},\varepsilon_{1},\cdots ,\varepsilon_{(M-1)/2},y(n-j-1),\cdots ,y(n-j-D)\right]\;\;\;(j=0,\cdots ,N)\;\varepsilon_{i}=\xi (n-j){\left|\xi (n-j)\right|}^{2i}\;\;\;(i=0,\cdots ,(M-1)/2)$$

Formula (10) can be solved by the least-squares method; then,

(11) $$h=U^{H}{\left(UU^{H}\right)}^{-1}y$$

Considering the impact of amplitude deviation,

$\xi (n)=\left(A_{m}+\Delta A\right)g(n)=\xi (n)+\Delta Ag(n)$, where g(n) is a rectangular pulse, ΔA is a constant, and ΔAg(n) can be considered as a constant A=ΔAg(n). The final effect is that there is a certain deviation between the amplitude of the actual signal and the amplitude of the reconstructed signal.

(12) y-A=Uh+V

We traverse *A* when *A* obtains optimal solution $A^{\prime }$, $A^{\prime }=\underset{A}{\arg\min}v(h)$, where v(h) represents the variance of solution feature h. After searching and solving $A^{\prime }$, Formula (11) is substituted to obtain an individual characteristic vector h of the radiation source. The final feature vector is $e=[A^{\prime },h]$.

### 2.3. Quadrature Modulation Distortion Mode

**Figure 3:**
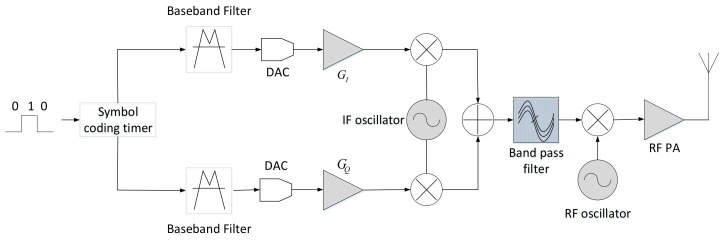
Phase-modulation flowchart.

The flow chart of quadrature-phase modulation is shown in Figure 3. The fingerprint characteristics of the radiation source usually come from modules composed of analog devices, so the analog circuit modules after the DAC module shown in the figure may generate the radiation-source fingerprint. The error factor of the in-phase/quadrature (I/Q) modulator includes the four following parts.

- I/Q gain imbalance

**Figure 4:**
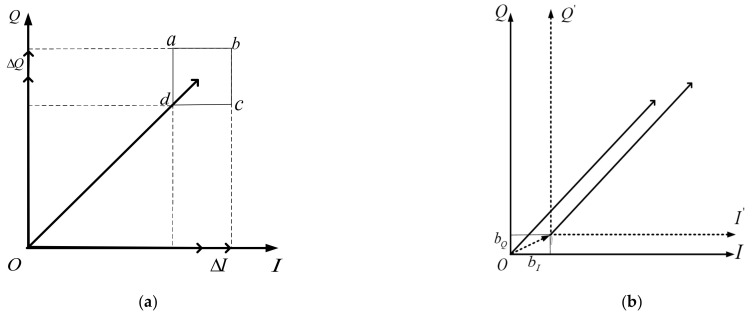
Phase deviation caused by modulator distortion. (**a**) Offset of phase center point caused by *I*/*Q* gain imbalance; (**b**) offset of origin of phase coordinate caused by DC offset.

This is mainly caused by the difference in the gain characteristics of the components on the I/Q two channels, which are represented as GI and GQ in Figure 4a; this leads to the phase point to change within the quadrilateral of the ‘abcd’ siege, as shown in Figure 4a.

- I/Q DC offset and carrier leakage

This is mainly caused by the DC offset generated by the amplifier on the I/Q path and the carrier leakage generated by the mixer; as shown in Figure 4, the DC offset causes the origin of the phase coordinate to drift and cause phase errors.

- I/Q delay mismatch

This is mainly due to the difference in the delay characteristics of the analog components (amplifiers, mixers) on the I/Q channel.

- I/Q quadrature error

When the local oscillator generates two orthogonal local carriers due to the characteristic error of the components, the phase difference is not 90°, that is, the quadrature error. These errors distort the constellation diagram on the demodulation constellation diagram, and the error characteristics of different individual transmitters are often different.

For the I/Q quadrature modulator, by gain imbalance $G_{m}=G_{I}/G_{Q}$, the DC offset of the I and Q channels is denoted by $b_{I}$ and $b_{Q}$, respectively. The delay mismatch is τ, and the quadrature error is denoted as ε. Then, I channel signal I(n) and Q channel signal Q(n) can be expressed as:

(13) $$I(n)=G_{m}\cos\left(2\pi \sum_{i}h_{i}d_{i}g(n-iT_{d})\right)+b_{I}$$

(14) $$Q(n)=\sin\left(2\pi \sum_{i}h_{i}d_{i}g(n-iT_{d}-\tau )\right)+b_{Q}$$

where h is the modulation coefficient, $d_{i}$ is the symbol sequence generated by the symbol encoder, and g(n) is the instantaneous pulse. The signal obtained by quadrature modulation is:

(15) $$x(n)=I(n)\cos(\omega_{c}n+\varepsilon /2)+Q(n)\sin(\omega_{c}n-\varepsilon /2)$$

Substituting Formulas (13) and (14) into Formula (15) produces:

(16) $$x(n)=x_{m}(n)e^{j2\pi fn}=e^{j2\pi f_{0}n}\left(u_{m}\xi (n)+v_{m}\xi^{*}(n)+b_{I}+b_{Q}\right)$$

where $f_{0}$ is the residual frequency offset, $\xi (n)=e^{j2\pi \sum_{i}h_{i}d_{i}g(n-iT_{d})}$; let $B=b_{I}+b_{Q}$ be the DC offset, and

(17) $$\begin{array}{l} u_{m}=\frac{1}{2}\left(G_{m}+1\right)\cos(\varepsilon /2)+\frac{1}{2}j\left(G_{m}-1\right)\sin(\varepsilon /2)\\ v_{m}=\frac{1}{2}\left(G_{m}-1\right)\cos(\varepsilon /2)+\frac{1}{2}j\left(G_{m}+1\right)\sin(\varepsilon /2) \end{array}$$

Combined with the Hammerstein model of the power amplifier, the effect of bias is not considered, $x(n)=u_{m}\xi (n)+v_{m}\xi^{*}(n)$, the equivalent discrete form of the output signal of the power amplifier is:

(18) $$\begin{array}{ll} y(n) & =\sum_{i=1}^{D}\alpha_{i}y(n-i)+\sum_{i=1}^{M}\lambda_{i}{\left(x(n)\right)}^{i}\\ & =\sum_{i=1}^{D}\alpha_{i}y(n-i)+\sum_{i=1}^{(M-1)/2}\frac{1}{2^{2i}}C_{2i+1}^{i+1}\lambda_{2i+1}\sum_{j=-i}^{i+1}\left(\sum_{k=0}^{i}C_{i+1}^{i+k}C_{i}^{k}\chi_{j+k,k}^{i+1-j-k,i-k}\right)\kappa_{i+j,i+1-j}^{n} \end{array}$$

where *D* and *M* are the AR order and polynomial order in the Hammerstein model, $\alpha =[\alpha_{1},\cdots ,\alpha_{D}]$; $\lambda =[\lambda_{1},\cdots ,\lambda_{M}]$ are the AR coefficient and polynomial coefficient, respectively, and, κ and χ are defined as follows:

$$\kappa_{a,b}^{n}={\left(\xi (n)\right)}^{a}{\left(\xi {\left(n\right)}^{*}\right)}^{b},\chi_{a,b}^{c,d}={\left(u_{m}\right)}^{a}{\left(u_{m}{}^{*}\right)}^{b}{\left(v_{m}\right)}^{c}{\left(v_{m}{}^{*}\right)}^{d}$$

Formula (18) can also be written as:

(19) q(n)=u(n)h+v

where

$$u_{n}=[\rho_{0}^{n},\cdots ,\rho_{M-1/2}^{n},y_{n}^{\prime }]\in {\mathbb{R}}^{1\times \left((M+1)(M+3)/4+D\right)}\;h={\left[\eta_{0},\eta_{1},\cdots ,\eta_{M-1/2},\alpha \right]}^{T}\in {\mathbb{R}}^{1\times \left((M+1)(M+3)/4+D\right)}\;\rho_{i}^{n}=[\kappa_{0,2i+1}^{n},\cdots ,\kappa_{j+i,i+1-j}^{n},\cdots ,\kappa_{2i+1,o}^{n}]\;\;\;(i=0,\cdots ,M-1/2)\;y_{n}^{\prime }=[y(n-1),\cdots ,y(n-D)]\;\eta_{i}=\frac{1}{2^{2i}}C_{2i+1}^{i+1}\lambda_{2i+1}\left[\chi_{0,i}^{i+1,0},\cdots ,\sum_{k=0}^{i}C_{i+1}^{i+k}C_{i}^{k}\chi_{j+k,k}^{i+1-j-k,i-k},\cdots ,\chi_{i+1,0}^{0,i}\right]\;\left(i=0,\cdots ,M-1/2,j=-i,\cdots ,i+1\right)$$

For observation period *N*, the above formula can be written in a concise matrix form:

(20) y=Uh+v

where

$$y={\left[y(n),\cdots ,y(n-N)\right]}^{T},U=\left[\begin{array}{c} u_{n}\\ \vdots \\ u_{n-N} \end{array}\right]$$

Considering the impact of DC bias, $x(t)=u_{m}\xi (t)+v_{m}\xi^{*}(t)+B$. Bias *B* is a real number, and the final effect is that a certain deviation exists between the amplitude of the signal that did not pass through the power amplifier, and the amplitude of the reconstructed signal. We traverse *B* when *B* obtains optimal solution $B^{\prime }=\underset{B}{\arg\min}v(h)$, where v(h) represents the variance of solution feature h. After searching and solving $B^{\prime }$, Formula (20) is substituted to obtain an individual characteristic vector h of the radiation source. The final feature vector is $e=[B^{\prime },h]$.

### 2.4. Integral Bispectrum

High-order spectral analysis is widely used in signal processing. In theory, a high-order spectrum can completely suppress any Gaussian noise and symmetrically distributed non-Gaussian noise. It can retain signal amplitude and phase information and is independent of time. Therefore, high-order spectral analysis is the current mainstream feature extraction method [11,26,27]. The third-order spectrum is the simplest high-order spectrum, also known as the bispectrum. Though the processing method is relatively simple, it can describe the nonlinear characteristics of the signal. For the digital zero-IF I/Q signal r(t) of the communication radiation source, its bispectrum can be defined as:

(21) $$B\left(\omega_{1},\omega_{2}\right)=\int_{-\infty }^{+\infty }\int_{-\infty }^{+\infty }c_{3r}\left(\tau_{1},\tau_{2}\right)e^{\left[-j\left(\omega_{1}\tau_{1},\omega_{2}\tau_{2}\right)\right]}{d\tau }_{1}{d\tau }_{2}$$

where $c_{3r}\left(\tau_{1},\tau_{2}\right)=E\left\{r^{*}\left(t\right)r\left(t+\tau_{1}\right)r\left(t+\tau_{2}\right)\right\}$ is the third-order cumulant of r(t).

**Figure 5:**
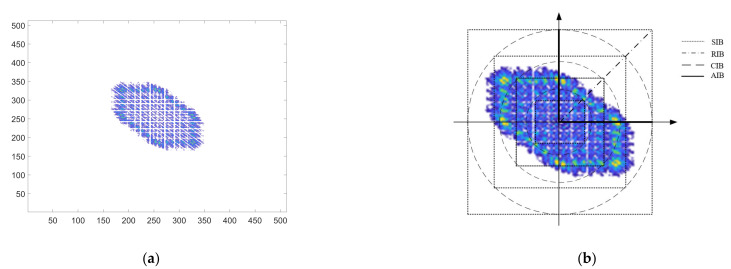
(**a**) Result of bispectral analysis of signal by Emitter 1 under signal-to-noise ratio of 15 dB. (**b**) Four paths for integrating two-dimensional bispectral features.

Compared with the power spectrum, it can provide phase information and is widely used. Integrating the bispectrum is the best way to reduce the dimensionality of two-dimensional bispectral features. According to different integration paths, it can be divided into RIB, AIB, SIB, and CIB. The integration path of each integral bispectrum is illustrated in Figure 5b.

## 3. Simulation and Experiment Results

### 3.1. Simulation Verification of PAM-Modulated Radiation Source

To verify the identification performance of the method on the ASK signal, the amplitude deviation of the transmitter and the nonlinearity of the power amplifier were simulated through a simulation signal. The first selection of $\lambda_{i}$ and α was determined by our demodulation and reconstruction of the real signal, and by fitting. Then, the subtle differences on the fitted data were randomly changed to become our four simulated radiation-source power-amplifier parameters, and amplitude error ΔA was added on this basis.

**Figure 6:**
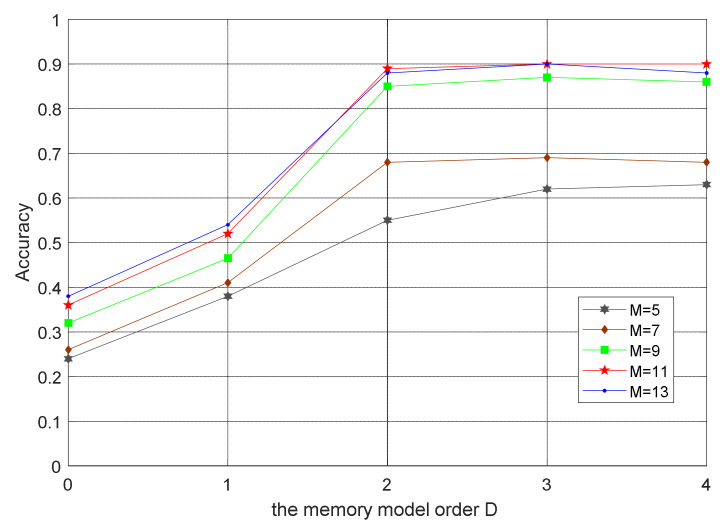
Influence of different model structures on accuracy.

The simulation signal constructed four radiation-source PAM signals with different parameters, five times oversampling; the radiation-source simulation parameter setting table is shown in Table 1. Figure 6 shows that polynomial order M of the nonlinear model of each radiation-source power amplifier and order D of the memory model was set to 11 and 2, respectively [1]. The number of signal samples of each radiation source was 1000, which were proportionally divided into training and test sets. By adding Gaussian white noise, the signal-to-noise ratio varied from 0 to 35 dB.

**Figure 7:**
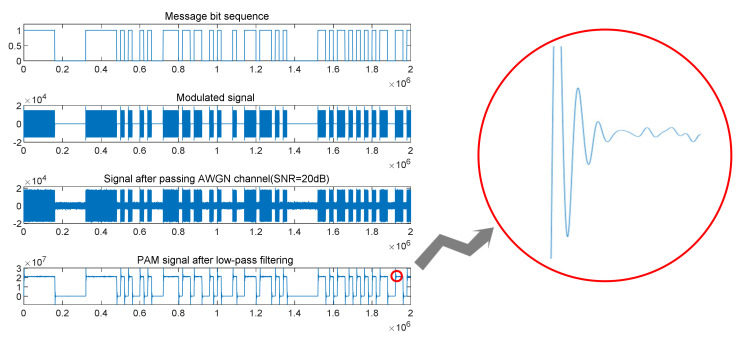
Signal simulation flowchart: entire signal process from original message bits to demodulation after passing through the channel; partial signal enlargement after low-pass filtering. Jitter reflects nonlinear characteristics of the power amplifier in the signal-amplification process.

We used software to simulate four simple PAM signals concerning the above nonlinear coefficients of the power amplifier. The simulated signal carrier was fc = 1 MHz, the information rate was 100 bit/s, and the sampling rate was five times the carrier frequency. The simulated signal is shown in Figure 7.

#### 3.1.1. Emitter Separability Analysis

The feature vector e of the radiation source was extracted using Formulas (12) and (20). We chose commonly used classifier algorithms by scholars, namely, convolutional neural network (CNN) and support vector machine (SVM). The distortion and nonlinear parameters were extracted were compared with the two classifiers.

Since extracted features e were only nine dimensions, we used three convolutional layers, two fully connected layers, and softmax layers to form our neural-network structure; the ‘padding’ method of the CNN was the same, the size of the convolutional kernel was 2 × 2, the stride was 1, pooling size was 2 × 2, and the stride was 1.

The SVM algorithm was originally designed for binary classification problems. When dealing with multiclass problems, it is necessary to construct a suitable multiclass classifier. At present, there are two main methods for constructing SVM multiclass classifiers: first, the direct method, which directly modifies the objective function, combines the parameter solving of multiple classification surfaces into an optimization problem and realizes multiclass classification by solving the optimization problem once. This method seems simple, but its computational complexity is relatively high, and it is relatively difficult to implement. It is only suitable for small problems.

Second, the indirect method, which mainly combines two classifiers to achieve the construction of multiple classifiers. The method is to design an SVM between any two types of samples, so k(k − 1)/2 SVMs need to be designed for *k* types of samples. When classifying an unknown sample, the category with the most votes is the category of the unknown sample. To classify four different radiation sources, we needed to design 6 SVM classifiers. The randomly selected training samples for each category were 800, and the test samples were the remaining 200. The kernel function of the SVM classifier was ‘rbf’, which is used to implement nonlinear classification.

**Figure 8:**
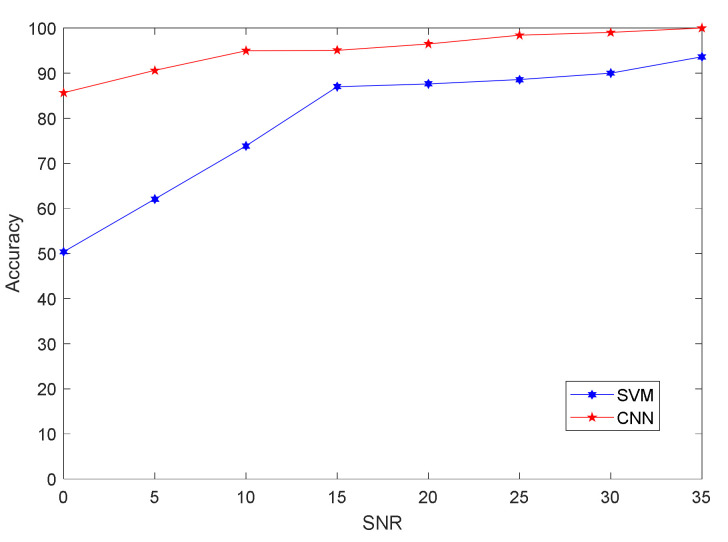
Accuracy of ASK signal features under different signal-to-noise ratio conditions through CNN and SVM classifiers.

Under different signal-to-noise ratio conditions, the recognition accuracy of the two classification methods is shown in Figure 8.

**Figure 9:**
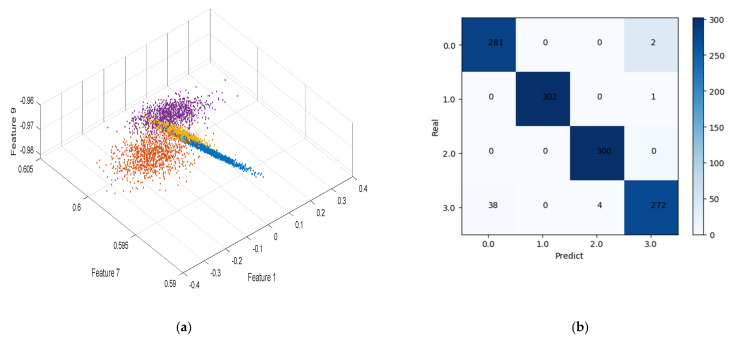
With a SNR ratio of 15 dB: (**a**) three-dimensional visualization map that composed 1-, 7-, and 9-dimensional data of feature vector e; (**b**) confusion matrix corresponding to CNN classification result.

As illustrated in Figure 8, the CNN classifier algorithm was generally better than the SVM classifier algorithm, and the CNN algorithm could more strongly distinguish with a low signal-to-noise ratio. When the signal-to-noise ratio was greater than 15 dB, the classification accuracy rates of the two classification algorithms, SVM and CNN, exceeded 80%. Figure 9a shows the three-dimensional visualization of the 1-, 7-, and 9-dimensional vectors of the individual characteristics of the four radiation sources extracted by the algorithm when the signal-to-noise ratio was 15 dB. Figure 9b is the confusion matrix given by CNN in this signal-to-noise ratio. Figure 9a shows that radiation sources 4 and 1 were classified incorrectly. Generally speaking, the feature vector obtained by this method could discriminate well.

#### 3.1.2. Comparison Experiment of Different Feature-Extraction Algorithms

In [7,10], the authors used the skewness and kurtosis algorithm to extract fingerprint features of a radiation source hidden in the signal. The authors in [10] used EMD, ITD, and VMD to decompose the signal, and then extracted the skewness and kurtosis information of each component as the fingerprint feature of the radiation source. In [26], the authors optimized skewness and kurtosis, and proposed a method for identifying individual characteristics of radiation sources based on linear skewness and linear kurtosis, which achieved a 100% distinction of three transmitters. The authors in [24] used the combination of an integral SIB bispectrum and a CNN to achieve a good recognition effect on five simulation data.

**Figure 10:**
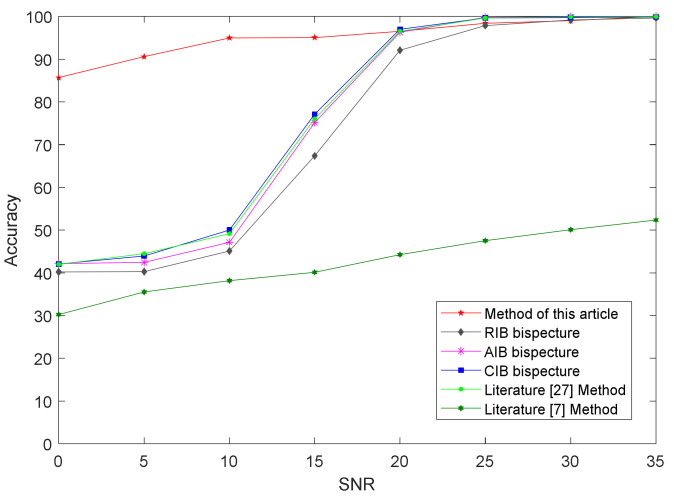
Recognition results of integral bispectrum algorithms, the algorithm in [27], and the proposed algorithm under different signal-to-noise ratio conditions.

Figure 10 shows the recognition-accuracy results of the proposed method and the integrated bispectrum through the CNN under different signal-to-noise ratio conditions. The figure shows that, when the SNR was less than 20 dB, the recognition rate of the proposed method was higher than that of the bispectral method. When the SNR was greater than 20 dB, the two methods had similar levels of recognition accuracy. This also shows that the bispectral method could achieve better recognition performance under a high signal-to-noise ratio.

#### 3.1.3. Changing Carrier Frequency

At present, individual radiation-identification methods use training-set signals to train the classifier, and then use signals with the same modulation parameters as the training set as the test set for testing. The actual radiation source can change the modulation parameters of the transmitted signal, such as changing the carrier frequency, modulation rate, and modulation mode of the transmitted signal. In this way, the traditional radiation-source identification method is no longer applicable because the classifier is not trained to change the modulation parameters of the signal. Changes in these modulation parameters make some existing research methods no longer have good robustness.

**Figure 11:**
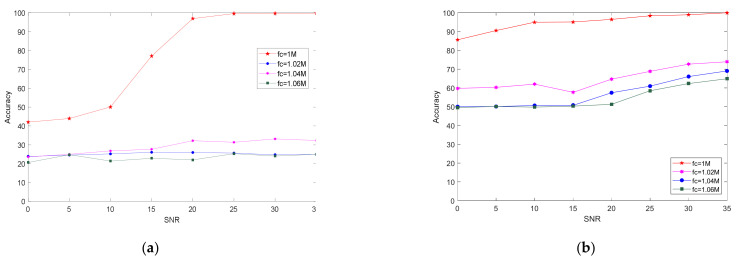
Experimental results when using different carrier-frequency data and (**a**) bispectral method or (**b**) proposed method as the test set.

The method in this paper extracts the distortion parameters of the inherent hardware of the transmitter, which does not change with a change in the carrier frequency, rate, and modulation mode of the transmitted signal. Therefore, it can recognize variable modulation parameters well. To verify the robustness of the proposed method under different parameter settings, we changed the carrier frequency of the test data. The carrier frequency of the training set was fc = 1 MHz, and the carrier frequency of the test set was set to fc = 1, 1.02, 1.04, and 1.06 MHz. Figure 11 shows the relationship between recognition accuracy and SNR after changing the carrier frequency of the test set using the proposed method and the dual-spectrum method. After the two methods changed the test set frequency, the recognition accuracy was reduced.

The specific reasons are analyzed as follows: for the integrated bispectral algorithm, the bispectral feature extracted information such as the frequency and phase of the signal. When using a test signal that was different from the carrier frequency of the training set, due to the test set signal frequency and the training set signal frequency, the difference was large. Although the signals were emitted by the same transmitter, their bispectral characteristics were quite different, which failed to achieve the purpose of identifying individual radiation sources. For the algorithm in this paper, changing different carrier frequencies, when we demodulated and reconstruct the signal, due to the error in the estimation of the carrier frequency and the estimation of the symbol rate, the reconstructed message signal had a certain error, which led to a decline in recognition accuracy.

**Figure 12:**
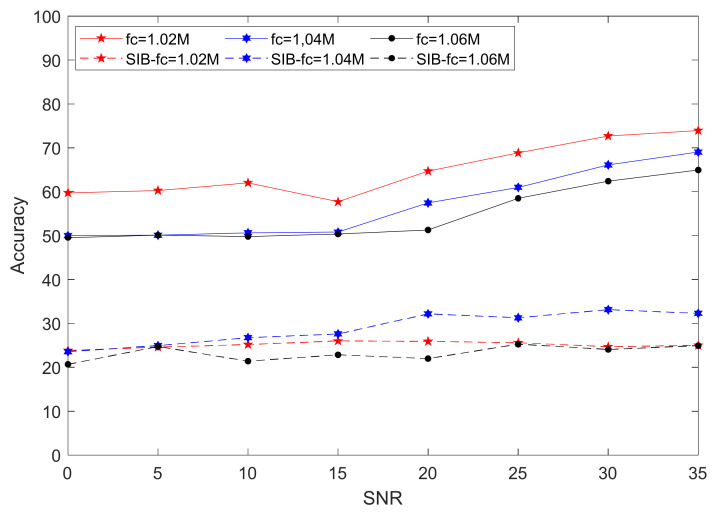
Relationship between recognition accuracy and SNR of the proposed method and bispectral method on test-set data of different carrier frequencies.

Figure 12 shows that, in the classification experiment of the four individual radiation sources, the SIB algorithm had a recognition accuracy of about 25% after changing the carrier frequency of the test set. The bispectral method appeared to not face the test signal with new modulation parameters to be able to classify individuals. The proposed method still had 50% accuracy when the signal-to-noise ratio was 0 dB. When the signal-to-noise ratio was high enough, recognition accuracy could exceed 70%. Figure 12 shows that, when the frequency of the test set was farther away than fc = 1 MHz, recognition accuracy showed a downward trend.

#### 3.1.4. Change in Information Rate

**Figure 13:**
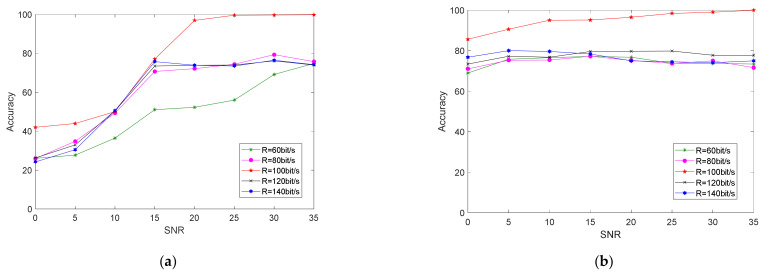
After changing the data information rate of the test set, the classification accuracy results using (**a**) bispectral method and (**b**) proposed method under different signal-to-noise ratio conditions.

To verify the robustness of the proposed method under different parameter settings, we changed the information rate of the test data. The information rate of the data we use for training was 100 bit/s, and the information rate of the test data was R = 60, 80, 100, 120, and 140 bit/s. Experiment results are shown in Figure 13 below.

**Figure 14:**
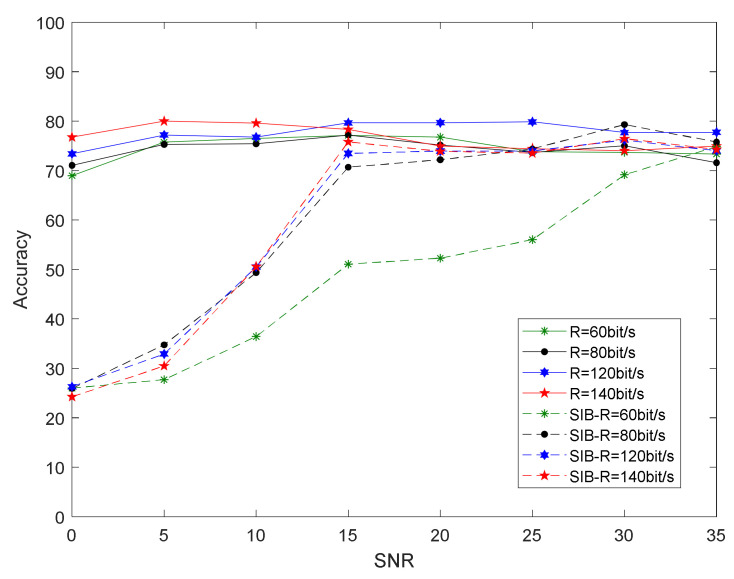
Classification-accuracy results of the proposed method and bispectral method under different SNR conditions after changing test-set information rate.

As shown in Figure 14, after changing the message rate of the test set, the recognition accuracy of the two methods decreased. When the signal-to-noise ratio was greater than 15 dB, the recognition accuracy of the two algorithms was maintained at 70–80%.

**Figure 15:**
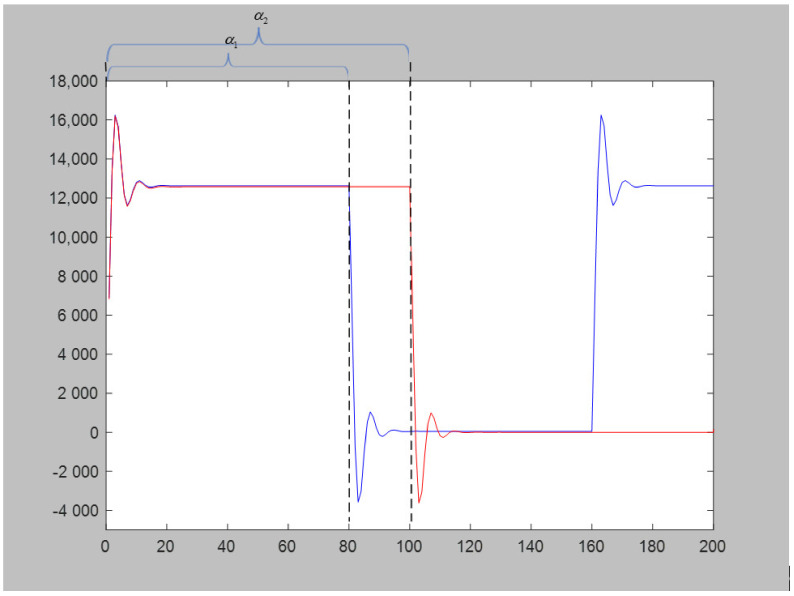
Baseband signal after passing through the power amplifier.

When changing the message rate, because the bispectral feature extracted the information of the signal frequency and phase, it had certain robustness to the change in message rate. Both algorithms had a certain drop in accuracy because, when the message rate changed, the length of the baseband shaping signal also changed. This means that the proportion of unstable points caused by the distortion of the modulator and the jitter caused by the nonlinearity of the power amplifier in the entire piece of data changed, as shown in Figure 15 (α1 and α2) The proposed method needs to use all data points to calculate the final feature vector *e*. Therefore, changing the message rate of the test set produced a certain error in the results.

### 3.2. Simulation and Verification of QPSK Modulated Radiation Source

To verify the ability of this method to identify QPSK signals, we simulated four QPSK signals, and the nonlinear parameters of the power amplifier were set as in the previous experiment. On this basis, unique gain imbalance $G_{m}$, DC offset, carrier leakage *B*, and quadrature error ε of the QPSK signal were added. Parameter settings are shown in Table 2.

**Figure 16:**
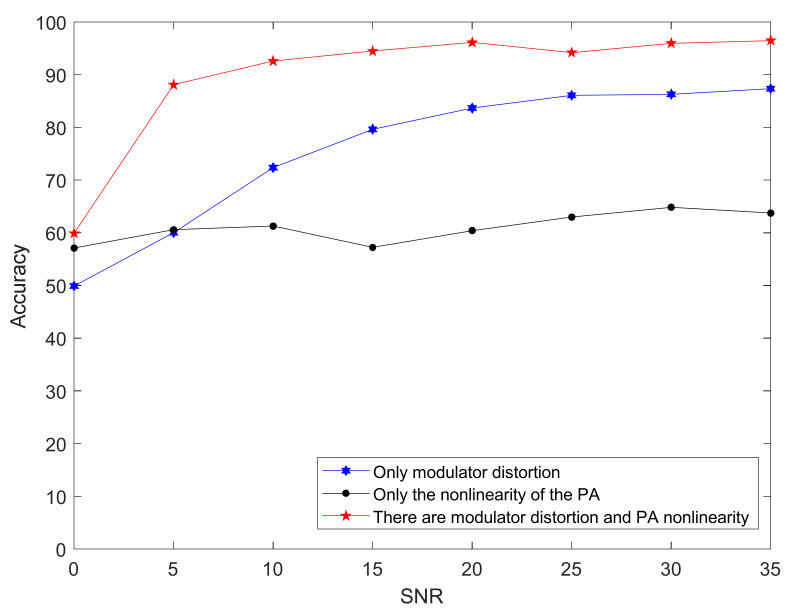
Classification-accuracy rate of three different models through CNN classifier under different signal-to-noise ratio conditions.

By setting the value of the parameter, we could obtain a model with only power nonlinearity, a model with only modulator distortion, and a model with both power amplifier nonlinearity and modulator distortion. Experiment results obtained are shown in Figure 16.

**Figure 17:**
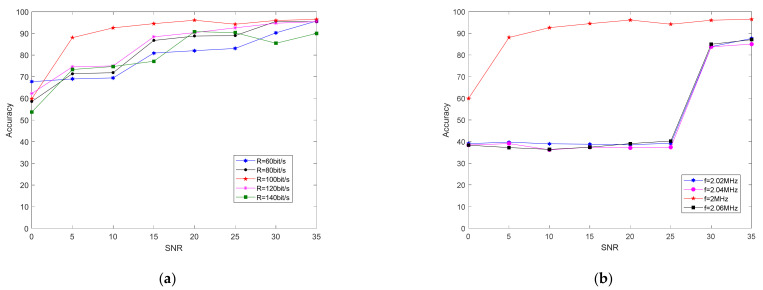
Classification-accuracy results of the proposed method under different signal-to-noise ratio conditions after changing (**a**) the data information rate and (**b**) the carrier frequency of the test set.

The Figure 17 shows that, when considering both the modulator distortion and the nonlinearity of the power amplifier, the model had the highest recognition rate under any SNR condition. When only considering the power amplifier, the accuracy rate did not change much with the increase in signal-to-noise ratio, indicating that the power amplifier did not have much influence on the signal because the QPSK signal was a constant envelope signal, and the phase of the signal was modulated. There was little correlation with the amplitude of the signal.

## 4. Discussion

By demodulating the digital modulation signal, the initial information sequence was obtained; then, by reconstructing the message signal, a signal without any modulation distortion and power amplifier nonlinearity were obtained. By comparing the deconstructed signal with the actual received RF signal through the feature-extraction algorithm, the parameter representation of the difference between the two could be obtained. The parameter that characterized the difference between the two was the feature vector that we needed, which can represent the difference between individual radiation sources. The proposed method puts forward the reason for the difference in a radiation-source signal rather than in the appearance. Through the extraction and identification of distortion parameters, the effective identification of individual radiation sources is achieved.

The traditional SEI idea is to use training-set signals to train the classifier, and then use the signal with the same modulation parameters as the training set as the testing signal. When the test- and training-set signals come from the same transmitter but have different modulation parameters, changes in these modulation parameters make some existing research methods no longer have good robustness. The proposed method extracts the distortion parameters of the inherent hardware of the transmitter, and the distortion parameters do not change with changes in carrier frequency and the rate of the transmitted signal. Therefore, it can identify variable modulation parameters well.

The characteristics of the transmitter extracted by the proposed method can represent the characteristics of the transmitter hardware. The characteristics do not change with the change in signal-modulation parameters (such as modulation mode, modulation rate, and carrier frequency) transmitted by the transmitter. The intelligent identification of a specific transmitter can then be realized.

## 5. Conclusions

This paper proposed a method for individual radiation-source identification based on modulator distortion and power-amplifier nonlinearity. This method can complete the extraction of transmitter hardware characteristics. Signals with different modulation rates and carrier frequencies from the same transmitter have robust recognition capabilities. It was experimentally verified that, when the signal-to-noise ratio was lower than 20 dB, the proposed feature method was better than the bispectral feature-extraction method. When the signal-to-noise ratio was 0 dB, the recognition ability of four different transmitters using the proposed feature-extraction method exceeded 85%. When the carrier frequency was changed, the signal characteristics extracted by the bispectral method could not distinguish individual transmitters. The proposed method could still achieve 70% accuracy in the identification of four different PAM transmitters when the signal-to-noise ratio was high enough. The identification of four different QPSK transmitters could achieve a correct rate of more than 80% when the signal-to-noise ratio was sufficiently high.

## Figures and Tables

**Table 1:** ASK-modulated radiation-emitter parameter setting.

Parameter	Emitter 1	Emitter 2	Emitter 3	Emitter 4
$\lambda_{1}$	1	1	1	1
$\lambda_{3}$	−42.38	−40.29	−44.57	−88.62
$\lambda_{5}$	2686.28	2409.38	2028.72	2242.64
$\lambda_{7}$	16,675	16,420	15,892	17,024
$\lambda_{9}$	−1652.42	−1589.88	−1892.46	−1686.27
$\lambda_{11}$	−5648.64	−6278.96	−7224.84	−5946.56
$\alpha_{1}$	1.9122	1.9108	1.9048	1.9143
$\alpha_{2}$	−0.9794	−0.9986	−0.9042	−0.9342
ΔA	0.1	0.15	0.08	0.1

**Table 2:** QPSK-modulated radiation-source parameter setting.

Parameter	Emitter 1	Emitter 1	Emitter 3	Emitter 4
$G_{m}$	0.9	1	1.2	1.3
B	0.1	0	0.12	0.05
ε	1°	0°	1°	0°

## Data Availability

Not applicable.
